# Ageing and Disability According to the Perspective of Clinical Psychology of Disability

**DOI:** 10.3390/geriatrics7030055

**Published:** 2022-05-10

**Authors:** Donatella R. Petretto, Roberto Pili

**Affiliations:** 1 Department of Education, Psychology and Philosophy, University of Cagliari, 09124 Cagliari, Italy; 2 Global Community on Longevity, 09124 Cagliari, Italy; dott.robertopili@gmail.com; 3 IERFOP Onlus, via Platone 1/3, 09100 Cagliari, Italy

The progressive ageing of the global population is an important anthropological and social phenomenon, and it is due to the overall increasing of life expectancy and the overall increasing of health and living conditions, even if with various trends and speeds in various countries all over the world. People can live longer than in the past and they can go through their entire lifespan, reaching the more advanced stages of possible human life [1,2]. However, the increasing of life expectancy does not correspond completely to the increasing of healthy life expectancy, and longer lives are associated with higher risk of developing age-related disorders and diseases and disability [3,4]. Thus, the progressive population ageing could lead to more individuals living the more advanced phase/phases of life with age-related diseases/disorders and with disability [1,2]. People with disability also have an increased life expectancy; they can live longer than in the past.

Considering this new situation, it may be very important to consider how to “add life to years” and how to promote wellbeing and good quality of life of individuals, even more if they have age-related disorders and/or disability [1,2,3]. Additionally, it may be very important to consider how to prepare people to “deal” with the entire life cycle from the earliest stages of life and how to help people to develop strategies and tools to “deal” with all the stages of life even with pathologies and disabilities. In fact, there is also an international agreement in the view that “ageing well” (active ageing or healthy ageing or positive ageing) is not without disorders or without diseases, but it refers to wellbeing and quality of life, even in the presence of a disease or a disorder [5,6,7,8,9,10].

So, it is important to consider how to help people to make use of what is currently possible from science and from technology to put in place to get to the best possible conditions in the more advanced stages of life. It is therefore important to understand how to ensure that people can live these advanced stages of life in conditions of well-being and good quality of life, with independence and autonomy. In other words, the fundamental question concerns: how we can learn to live for a long time and, possibly, live well and with independence and autonomy.

In this editorial, we aim to address the relationship between ageing and disability according to the perspective of Clinical Psychology of Disability. Clinical Psychology of Disability may have a role in the different aspects, domains, and steps of the life of ageing people with disability in each phase of life and in the support of people with disability who age and go through the more advanced phases of life. It may have a role in the assessment of functional profile, in the diagnosis, in the coping, in the empowering and in the support of people with disability in each phase of life and in the support people with disability who age and go through the more advanced phases of life. According to a life course perspective, there can be two main developmental trajectories in the relationship between ageing and disability: the first trajectory is named “ageing with disability” and it refers to individuals with disability who have peculiar health conditions since the first step of life (infancy, adolescence, and adulthood) and that, thanks to the overall increase in life expectancy, can live longer than in the past; the second trajectory is named “disability with ageing” and it refers to individuals that, thanks to the overall increase in life expectancy, live longer than in the past and they develop age-related health conditions correlated to disability only in the more advanced phases of life [3,4,11,12]. Some other authors used a different terminology, for example “ageing with disability” and “ageing into disability” [13].

According to the perspective of Clinical Psychology of Disability, in each trajectory, the individual faces developmental transitions, develops identity and acquires roles, but there can be great differences in these aspects according to the time when individuals develop health conditions and meet disability (earlier in the first trajectory and later in the second trajectory). Moreover, in each trajectory, the individual acquires information about his/her health condition/diagnosis and about his/her functional profile; he/she develops coping strategies and adjustment abilities, and faces life events. However, there can be great differences in these aspects according to the time when individuals develop health conditions related to disability (earlier in the first trajectory—sometimes so early that some aspects and experiences are directly mediated by parents or other family members, such as when it comes in infancy or in the first adolescence—and later in the second trajectory). With reference to a life project, the trajectories can differently influence continuity and discontinuity in autobiographical paths, and in general life paths; moreover, they differently impact on autobiographical self and autobiographical consciousness. Furthermore, these two different trajectories can interact in a different way with/and affect the needs, expectations, and life project of everyone.

Taking into consideration all these aspects, it becomes important to pay attention to each of these trajectories individually, and therefore to consider the experiences of people who live these developmental trajectories, highlighting the differences and specificities but also the points of contact and elements of convergence between the two trajectories. Because the increase in life expectancy is, however, a recent phenomenon, both trajectories and, again, the experiences of the people who live these developmental trajectories, also represent a field of particular interest as they are very recent phenomena [1,2,3,4].

1. Some Premises and Lines of Analysis in the Study of Relationship between Ageing and Disability with the Perspective of Clinical Psychology of Disability

The relationship between ageing and disability is a complex one for several reasons.

First, because as already mentioned there can be two fundamental developmental trajectories well-described by the terminology “ageing with disability” and “disability with ageing”. These two trajectories present interesting specificities from the point of view of Clinical Psychology of Disability and it is important to deepen each of these trajectories individually, highlighting the differences and specificities but also analyzing the interest towards contact points and elements of convergence between the two trajectories.

In addition, the relationship between ageing and disability is a complex one in that both the concepts of ageing and disability refer to dynamic processes related to a continuous interaction between the person and the environment. Let us clarify this aspect better in both concepts and in the relationship between them. According to a biopsychosocial point of view, ageing is a complex phenomenon and is a physiological process, a psychological process, and a social process; it is a stage of the life cycle, the most advanced stage, or stages [5,6,7,8,9,10,14,15,16]. This complex phenomenon can be analyzed from a biological, psychological, and social point of view, with a biopsychosocial perspective that aspires to integrate these different levels, even if it is not a simple task. Elsewhere, we have also considered biological and genetical level [1,2,17]. From a psychological point of view, ageing is characterized by developmental tasks, transitions and continuity, identities, roles, and relationships. The most advanced stages of life represent an important step in the life cycle, a source of both challenges and opportunities, new learning, changes, losses, reorganizations but also continuity, adjustments, and novelties. This phase is characterized by roles, relationships, and activities that can be strongly influenced culturally, socially, and historically. It is a phase that can be characterized by continuity or discontinuity with respect to roles and tasks, and this can affect the autobiographical self and extended consciousness [18]. Ageing can also be characterized by a transition from working life, although in some social and cultural contexts this transition never occurs [17,18]. Like all phases of the life cycle, it involves challenges and opportunities. It involves all the different domains of the functioning of the person, from the cognitive one, to the socio-relational one, to the affective one and relative to the control of the emotions, as well as the motor and perceptive-sensory domains. In addition, it involves experiencing life events (positive and negative) and it involves forms of new development and new learning, and therefore may require the development and/or adaptation of previous resources and coping strategies. The number of life events is surely greater in those who face the more advanced stages of the life cycle: the longer life expectancy increases, in statistical terms, the probability of encountering life events (some of them negative or “subjectively” negative, such as bereavement, loss, reorganization, etc.). There may be factors of protection, risk, and vulnerability at the individual, family, and relational level, and at other micro- and macrosocial levels (family network, friendships, wider social network, formal and informal networks). From this point of view, it is important to consider that this process acts at the individual level but always involves relational and social aspects (family and family network, and/or other formal and informal social networks) and that there is a continuous interaction between the individual and the environment (relational, social, and cultural aspects of environment and the environment considered in the broad sense) [1]. In this context, it may be useful to consider two facets/processes of ageing, which are also in continuous interaction: primary ageing and secondary ageing. Primary ageing is a genetically and biologically programmed process, still uncontrollable and irreversible, linked to a possible progressive more or less rapid and more or less intense deterioration of physical/sensorial/cognitive/perceptual functions. Primary ageing may involve a possible and progressive functional loss at the cognitive, sensory, perceptive, and motor level, during the most advanced phases of life. Secondary ageing is intertwined with the former, but it could be subjected to forms of control/slowdown based on people’s behavior—lifestyle, diet, nutrition, relationships, and stress management, but it could also be affected by social and cultural factors, and environmental factors in a broad sense [2,17,18]. In the specific theme of the relationship between ageing and disability according to the Clinical Psychology of Disability, these facets/processes of ageing must be analyzed in their specificities and points of contact between the two different developmental trajectories described above (ageing with disability and disability with ageing) and it is necessary to take into account, for example, the possible speeding up of the ageing process that can occur due to the effect of the interweaving between different age-related pathologies and/or previous diseases and/or disabling health conditions, and that could be an interesting example of secondary ageing.

Disability is a complex phenomenon and a process, too; it is a process in which, according to the most recent and shared conceptual models in this field, disability itself is the negative “result” of the interaction between the individual with his or her pathologies and/or health conditions and abilities, and the environment [19,20,21]. From this point of view, a clear central role of social and environmental factors in this dynamic process is recognized. In conceptual models of disability, in a positive way, social and environmental factors can enable people with disabilities and optimize opportunities for health, well-being, participation, autonomy and independence; negatively, social and environmental factors can disable people and reduce their opportunities for health, well-being, participation, autonomy, and independence [20,21].

In the specific theme of the relationship between ageing and disability according to the Clinical Psychology of Disability, the complexity of the relationship between ageing and disability and the dynamicity of both concepts (ageing and disability) must be analyzed in a deeper way considering their specificities and points of contact between the two different developmental trajectories described above (ageing with disability and disability with ageing) and therefore the experiences of people who live these developmental trajectories [11,12]. In both trajectories, and again, in the experiences of the people who live these developmental trajectories, the social and environmental factors can act as a facilitator or as a barrier. Once again, in a positive way, social and environmental factors can promote the good and long life of people with disabilities or those who encounter disability in the most advanced stages of their lives, and can optimize their health opportunities, well-being, participation, autonomy, and independence; in a negative way, social and environmental factors can disable elderly people with disabilities or those who encounter disability at later stages of their lives and reduce their health opportunities, well-being, participation, autonomy, and independence.

2. Conclusions

In this editorial, we addressed some aspects of the complex relationship between ageing and disability according to the perspective of Clinical Psychology of Disability. We proposed and discussed some general principles and some lines of analysis, and we addressed some peculiarities and features of the two main developmental trajectories in this field. More research is needed in the description of the specific experience of people with disability that go through each trajectory, to highlight needs and rights. Moreover, more research is also needed to better clarify the role of Clinical Psychology of Disability in the promotion of empowerment, inclusion, and participation of people with disability who age, and in the development of intervention aimed to reduce barriers and to support people. From this regard, it is therefore a priority to gain evidence and knowledge and to share knowledge and awareness that allow people to be able to “equip themselves” with everything that may be necessary for this. Moreover, it is also a priority to inform institutions and societies on how to create and to guarantee the conditions for extending this opportunity to the highest possible number of people.
